# Influence of tumor extent on central lymph node metastasis in solitary papillary thyroid microcarcinomas: a retrospective study of 1092 patients

**DOI:** 10.1186/s12957-017-1202-8

**Published:** 2017-07-17

**Authors:** Xingjie Yin, Chunping Liu, Yawen Guo, Xiaoyu Li, Na Shen, Xiangwang Zhao, Pan Yu, Shan Wang, Zeming Liu

**Affiliations:** Department of Breast and Thyroid Surgery, Union Hospital, Tongji Medical College, Huazhong University of Science and Technology, Number 1277, Jiefang Road, Wuhan, Hubei Province China

**Keywords:** Solitary papillary thyroid microcarcinomas, Capsule invasion, Extrathyroidal extension, Central lymph node metastasis, Central lymph node dissection

## Abstract

**Background:**

The morbidity of papillary thyroid microcarcinomas is increasing worldwide. Surgery is the main treatment for papillary thyroid microcarcinomas, and the choice of surgical method partly depends on the T stage of the tumor. However, according to the American Joint Commission on Cancer staging system (7th edition), the T stage of papillary thyroid microcarcinomas with different tumor extent is unclear. We aimed to study the effect of tumor extent and other factors on central lymph node metastasis to explore the relationship between tumor extent and T stage and to identify the risk factors predicting central lymph node metastasis in papillary thyroid microcarcinomas.

**Methods:**

We included 1092 patients diagnosed with solitary papillary thyroid microcarcinomas between July 2011 and April 2016. The tumor extent and other central lymph node metastasis risk factors were retrospectively analyzed.

**Results:**

Univariate analysis revealed that capsule invasion and extracapsular extension (*P* = 0.013, <0.001; respectively) were significantly correlated with central lymph node metastasis. On multivariate analysis, extracapsular extension was independent central lymph node metastasis predictors (odds ratio 3.092, 95% CI 1.744–5.484), while capsule invasion was not (odds ratio 1.212, 95% CI 0.890–1.651). In addition, multivariate analysis revealed that male sex, tumor size >5 mm, and age <45 years were independent central lymph node metastasis predictors (odds ratio 2.072, 2.356, 2.302; 95% CI 1.483–2.894, 1.792–3.099, 1.748–3.031; respectively).

**Conclusions:**

This study supported that capsule invasion and tumor limited to the thyroid in papillary thyroid microcarcinomas were suitable for the lower T1, that is, capsule invasion in papillary thyroid microcarcinomas might not belong to the minimal extrathyroid extension included in T3 of TNM staging. In addition, patients with risk factors of extrathyroid extension, male sex, age <45 years, or tumor size >5 mm in papillary thyroid microcarcinomas should consider a more aggressive surgical treatment.

## Background

Papillary thyroid microcarcinoma (PTMC) is defined by the World Health Organization as a malignant tumor with diameter ≤1 cm [1]. In recent years, owing to the wide application of high-resolution and contrast-enhanced ultrasonography (US) of the thyroid, as well as ultrasonography-mediated fine needle aspiration biopsy (FNA), along with improved histopathological criteria, increasing numbers of patients have been diagnosed with PTMC [2, 3]. According to the current literature, the prevalence of solitary PTMC, that is, a single lesion in the thyroid gland, ranges between 18 and 79.7% among PTMC [4–6]. The thyroid capsule is composed of varying proportions of fibrous and adipose tissues and is discontinuous in the anterior midline of the isthmus [7]. As described in the American Joint Commission on Cancer staging system (7th edition), the T stage of PTMC varies with the relationship between the tumor and the thyroid capsule: tumor limited to the thyroid is T1a while PTMC with minimal extrathyroid extension (e.g., extension to sternothyroid muscle or perithyroid soft tissues) is T3. However, there are three cases of their relationship in clinical work: tumor limited to the thyroid (including primary tumor adjacent to the thyroid capsule), capsule invasion (no capsular penetration), and capsular penetration with extension into the sternothyroid muscle or perithyroidal soft tissue (extrathyroidal extension). Then, whether capsule invasion of PTMC belongs to T1a or T3 and whether we should choose the same operation for PTMC with capsule invasion and extrathyroidal extension is a problem that we clinicians are confused.

Meanwhile, the treatment of PTMC may vary from follow-up to a total thyroidectomy and lymph node dissection with or without radioactive iodine treatment, and the choice of surgical method partly depends on the T stage of the tumor. Although PTMC is believed to be an indolent disease, the reported incidence of central lymph node metastasis (CLNM) associated with PTMC has been reported to be as high as 41.3% [8], and CLNM is known to be related to poor prognosis and locoregional recurrence [9]. However, prophylactic central lymph node dissection (CLND) has been reported to increase postoperative morbidity (transient hypocalcemia or recurrent laryngeal nerve paralysis) [10, 11], and the use of total thyroidectomy (TT) plus CLND is thus debated for PTMC, because prophylactic CLND may have little prognostic benefit and increase postoperative morbidity [12]. Accordingly, it is important to identify the risk factors for CLNM so that appropriate clinical decisions regarding CLND can be made.

With this in mind, the present study was designed to explore whether capsule invasion belongs to the minimal extrathyroid extension and identify the risk factors associated with CLNM in PTMC by retrospectively investigating the effect of tumor extent and other factors on central lymph node metastasis in PTMC.

## Methods

### Patients

This was a retrospective study of patients treated at the Department of Breast and Thyroid Surgery, Union Hospital, Tongji Medical College, Huazhong University of Science and Technology, Wuhan, China, from July 2011 to April 2016. This study was approved by the institutional review board of the Union Hospital, Huazhong University of Science and Technology.

Patients were enrolled in the study if they fulfilled all of the following criteria: (i) no previous thyroid surgery; (ii) no neck lymph node metastasis on preoperative assessment (palpation, ultrasonography, and fine needle aspiration biopsy); (iii) TT plus bilateral prophylactic CLND was performed for treatment; and (iv) a diagnosis of primary solitary PTMC was made on final pathological examination. Patients with primary tumors in the isthmus of the thyroid gland were excluded. We excluded multicenter PTMC because the tumor extent of each single lesion might be different. As a result, a total of 1092 patients were enrolled in this study. Almost all patients were from central China, and most of them were from Hubei Province.

### Surgical treatment

Diagnostic preoperative workup included clinical examination and neck and thyroid ultrasonography with or without contrast-enhanced ultrasonography. Ultrasonography-mediated fine needle aspiration biopsy was not performed routinely. During the past few years in our unit, local recurrence was commonly found among PTMC patients who had undergone partial thyroidectomy. Therefore, during the study period, we performed total thyroidectomy associated with bilateral central neck dissection for patients diagnosed with malignancy, regardless of disease stage or size. During this period, the false-negative rate for our intraoperative frozen sections on PTMC averaged 3.9%, according to data collected by our department of pathology.

### Clinicopathological parameters

We analyzed tumor extent and the following variables as possible risk factors for CLNM in patients with solitary PTMC: sex, age at pathological diagnosis, tumor size, histological subtype of the PTMC, lymphocytic thyroiditis (lymphocytic thyroiditis includes “Hashimoto’s thyroiditis,” “lymphocytic thyroiditis,” or “chronic lymphocytic thyroiditis.” These terms represent a pathophysiologic spectrum of disease severity in our department). The extent of the primary thyroid tumor was categorized as follows: limited to the thyroid (including primary tumor adjacent to the thyroid capsule), capsule invasion (no capsular penetration), and capsular penetration with extension into the sternothyroid muscle or perithyroidal soft tissue.

### Statistical analysis

Continuous variables are expressed as the mean ± standard deviation. The chi-squared test was used for univariate analysis of the statistical correlations between the clinicopathological parameters and CLNM, while multivariate statistical analysis was performed using binary logistic regression to assess the independent associations between CLNM and the factors that were statistically significant in the univariate analysis. We calculated the odds ratio (OR) and 95% confidence interval (CI) using binary logistic regression. Statistical analyses were performed using SPSS Software version 19.0 (SPSS, Chicago, IL, USA). All statistical tests were two-sided, and *P* < 0.05 was considered statistically significant.

## Results

The clinicopathological characteristics of the 1092 enrolled patients are listed in Table 1. The mean age was 45.30 ± 10.33 years (range, 19–87 years), and there were 900 women (82.4%) and 192 men (17.6%). The median tumor size was 0.53 ± 0.23 cm (range, 0.05–1 cm); the primary tumor size was ≤5 mm in 602 patients and >5 mm in 490 patients. The tumor was located within the thyroid capsule in 699 (64.0%) cases, had invaded the capsule in 355 (30.7%) cases, and had penetrated the capsule and extended into the sternothyroid muscle or perithyroidal soft tissue in 58 (5.3%) cases. Lymphocytic thyroiditis was found in 129 patients. CLNM was found in 300 (27.4%) patients (Table 1), while contralateral lymph node metastasis accounted for 21.7% in patients with CLNM (data not shown).

**Table 1:** Clinicopathological characteristics of patients with solitary papillary thyroid microcarcinomas (*n* = 1092)

Parameter	Number (%) of patients (*n* = 1092)
Age (years)	
<45	513 (47.0%)
≥45	579 (53.0%)
Sex	
Male	192 (17.6%)
Female	900 (82.4%)
Size (mm)	
≤5	602 (55.1%)
>5	490 (44.9%)
Tumor extent	
Within capsule	699 (64.0%)
Capsule invasion	335 (30.7%)
Extrathyroidal extension	58 (5.3%)
Lymphocytic thyroiditis	
Present	129 (11.8%)
Absent	963 (88.2%)
Subtype	
Classic	1079 (99.8%)
Follicular	13 (1.2%)
CLNM	
Yes	300 (27.4%)
No	792 (72.6%)

*CLNM* central lymph node metastasis

According to the univariate analysis (Table 2), the tumor extent was significantly correlated with CLNM (*P* < 0.001). Male sex, age <45 years, and tumor diameter >5 mm were also significant predictive factors for CLNM (*P* < 0.05). In contrast, the histological subtype did not predict CLNM in solitary PTMC. Meanwhile, lymphocytic thyroiditis was revealed to be a protective factor against CLNM in patients with solitary PTMC.

**Table 2:** Associations between central lymph node metastasis (CLNM) and clinicopathological characteristics in solitary papillary thyroid microcarcinomas

Parameter	CLNM−	CLNM+	*P* value
	(*n* = 792)	(*n* = 300)	
Age (years)			
<45	321 (62.6%)	192 (37.4%)	
≥45	471 (81.3%)	108 (18.7%)	<0.001
Sex			
Male	111 (57.8%)	81 (42.2%)	
Female	681 (75.7%)	29 (24.3%)	<0.001
Size (cm)			
≤0.5	484 (80.4%)	118 (19.6%)	
>0.5	308 (62.9%)	182 (37.1%)	<0.001
Tumor extent			
Within capsule	533 (76.3%)	166 (23.7%)	
Capsule invasion	231 (69.0%)	104 (31.0%)	
Extrathyroidal extension	28 (48.3%)	30 (51.7%)	<0.001
Lymphocytic thyroiditis			
Present	104 (80.6%)	25 (19.4%)	
Absent	688 (71.4%)	275 (28.6%)	0.028
Subtype			
Classic	783 (69.2%)	296 (30.8%)	
Follicular	9 (69.2%)	4 (30.8%)	0.789

The analysis of the impact of the tumor extent on CLNM, with tumors limited to the thyroid as the reference, is shown in Table 3. The analysis showed that the risk of developing CLNM increased with capsule invasion (OR, 1.446; 95% CI, 1.082–1.931) and extracapsular extension (OR, 3.440; 95% CI, 1.997–5.925). Multivariate analysis revealed that while capsule invasion (*P* = 0.223) was not a statistically significant predictor of CLNM, extracapsular extension (*P* < 0.001) was indeed a statistically significant independent risk factor. In addition, male sex (OR, 2.072; *P* < 0.001), tumor size >5 mm (OR, 2.356; *P* < 0.001), and age <45 years (OR, 2.302; *P* < 0.001) were independent predictors of CLNM (Table 4).

**Table 3:** Risk of central lymph node metastasis in solitary papillary thyroid microcarcinomas according to tumor extent

Variable	Adjusted OR	95% CI	*P* value
Within capsule	1 (ref)		
Capsule invasion	1.446	1.082–1.931	0.013
Extracapsular extension	3.440	1.997–5.925	<0.001

*OR* odds ratio, *CI* confidence interval

**Table 4:** Multivariate logistic regression analysis of central lymph node metastasis in solitary papillary thyroid microcarcinomas

	Sig.	Exp (B)	95% CI	
			Lower	Upper
Age (<45 years)	<0.001	2.302	1.748	3.031
Sex (male)	<0.001	2.072	1.483	2.894
Size (>5 mm)	<0.001	2.356	1.792	3.099
Tumor extent				
Capsule invasion	0.223	1.212	0.890	1.651
Extrathyroidal extension	<0.001	3.092	1.744	5.484

*Sig.* significance, *Exp (B)* odds ratio for central lymph node metastasis, *CI* confidence interval

Among 1092 patients, transient hypocalcemia developed in 12 patients (1.09%) and 1 patients (0.09%) had permanent hypoparathyroidism. Recurrent laryngeal nerve injury developed in 17 patients (1.56%) and recovered within 6 months, and no cases experienced permanent recurrent laryngeal nerve paralysis. Chyle leakage occurred in 2 patients (0.2%). Chyle leakage was controlled with a fat-free diet and local compression. Postoperative hematoma developed in 5 patients (0.5%) and was controlled by reoperation.

## Discussion

According to the American Joint Commission on Cancer staging system (7th edition), in papillary thyroid microcarcinomas, T1a stage tumors are defined as tumors ≤1 cm without extrathyroidal extension, while any tumors with minimal extrathyroid extension (e.g., extension into the sternothyroid muscles or perithyroidal soft tissues) are classified as T3 stage tumors. However, it has not been decided whether PTMC with capsule invasion should be classified as stage T1a or T3. Because the recommendations for clinical treatment vary according to the TNM stage, accurate T categorization is very important. Several recent studies have described the association of capsule invasion and/or extracapsular extension with CLNM in patients with PTMC [6, 13]; in comparison, our study focused only on solitary PTMC and contained the largest number of patients and all patients underwent TT and CLND.

In the present study, multivariate analysis showed that extrathyroidal extension was independent predictor of CLNM in solitary PTMCs. Previous studies have also reported that extrathyroidal extension was associated with CLNM in patients with PTMC [3, 6, 13, 14]. Our study reveal that capsule invasion and tumor limited to the thyroid were not identified as independent risk factor for CLNM in solitary PTMC; this might indicate that capsule invasion does not belong to the minimal extrathyroid extension category and that tumors ≤1 cm with capsule invasion might be classified as T1a.

The incidence of CLNM in patients with clinically node-negative PTMC has been reported from 31 to 64% [6, 13, 15], and the presence of CLNM is closely associated with prognosis and is a key factor for tumor staging [9, 16]. However, the use of routine prophylactic CLND for PTMC is controversial due to the high risk of postoperative morbidity and the limited prognostic benefits [9, 17]. Moreover, CLNM cannot be detected clinically because of the local anatomy of the level VI lymph nodes [16]. In our study, 300 patients had cervical lymph node metastases, representing 27.5% of all 1092 patients, which was lower than previous studies [6, 13, 15]. This large range in the prevalence of CLNM may be due to differences that we focused on solitary PTMC only. In this study, we found that extrathyroidal extension, male sex, age <45 years, and tumor size >5 mm were risk factors for CLNM.

Younger age (age <45 years) was associated with CLNM in this study. However, it is unclear about its prognostic value in PTMC [13, 18]. Previous studies have also reported that age <45 years was associated with CLNM in PTMC [6]. Generally, CLNM is known to increase with tumor size, and tumor size >5 mm was an independent predictor of CLNM in my research, which is in agreement with a previous report [15]. In addition, consistent with some previous researches [6, 13, 15], gender was a risk factor for CLNM in my study.

Moreover, lymphocytic thyroiditis was shown to protect patients with solitary PTMC against CLNM in the univariate analysis, which was consistent with the findings of previous studies on papillary thyroid carcinoma [19, 20]. This may be explained from the following two aspects [21]: On one hand, the autoimmune response may destroy cancer cells expressing thyroid-specific antigens, thereby preventing cancer progression. On the other hand, if lymphocytic thyroiditis leads to atrophy and fibrosis of the thyroid gland, there may be associated damage to the surrounding lymphatic vessels, which may prevent lymphatic spread and reduce the likelihood of CLNM [20].

The treatment of PTMC is a contentious subject. In this study, our results supported the notion that a more radical surgical treatment is appropriate treatment strategy for PTMCs with extrathyroidal extension, male sex, tumor size, and age <45 years. This conclusion was based on the following considerations: first, as preoperative assessment is limited, bilateral cancer and multifocality of the thyroid might be assessed as a single focus; second, among patients with CLNM, contralateral lymph node metastasis accounted for 21.7% in our study. Morbidity of complications is low when TT plus CLND is performed by experienced surgeons in this study and CLND did not increase permanent morbidities obviously [14]. In addition, morbidity is relatively high when reoperation is performed on patients with regional recurrence in the central compartment. Moreover, TT plus CLND is beneficial for postoperative follow-up and radioactive iodine (131I) thyroid remnant ablation and is associated with a low recurrence rate. Thus, a more radical surgical treatment should be considered for PTMCs with high-risk factors.

## Conclusion

In conclusion, extrathyroid extension is a significant predictor of CLNM in PTMC. Our findings suggest that capsule invasion may not belong to the minimal extrathyroid extension category. Meanwhile, our results are useful in identifying PTMC with extrathyroid extension, male sex, tumor size >5 mm, and age <45 years, which are more likely to have CLNM, and on the basis of this, we could choose to perform more radical treatment in patients with potentially aggressive tumors. In addition, our study revealed that coexistent lymphocytic thyroiditis protects patients with PTMC from CLNM.

## Abbreviations

CI: Confidence interval
CLND: Central lymph node dissection
CLNM: Central lymph node metastasis
OR: Odds ratio
PTMC: Papillary thyroid microcarcinoma
TT: Total thyroidectomy
