# Correction to: Influence of tumor extent on central lymph node metastasis in solitary papillary thyroid microcarcinomas: a retrospective study of 1092 patients

**DOI:** 10.1186/s12957-017-1234-0

**Published:** 2017-10-17

**Authors:** Xingjie Yin, Chunping Liu, Yawen Guo, Xiaoyu Li, Na Shen, Xiangwang Zhao, Pan Yu, Shan Wang, Zeming Liu

**Affiliations:** Department of Breast and Thyroid Surgery, Union Hospital, Tongji Medical College, Huazhong University of Science and Technology, Wuhan, 430022 China

## Erratum

After publication of the original article [1] it was noted that the affiliation address had been incorrectly submitted by the authors.

The address affiliation address listed currently reads “*Department of Breast and Thyroid Surgery, Union Hospital, Tongji Medical College, Huazhong University of Science and Technology, Number 1277, Jiefang Road, Wuhan, Hubei Province, China”.*

At present the correct address is *“Department of Breast and Thyroid Surgery, Union Hospital, Tongji Medical College, Huazhong University of Science and Technology, Wuhan 430022, China”.*

This error had since been acknowledged and corrected in this correction article. The authors also wish to apologize for any inconvenience caused.
